# Effect of Breadmaking Process on *In Vitro* Gut Microbiota Parameters in Irritable Bowel Syndrome

**DOI:** 10.1371/journal.pone.0111225

**Published:** 2014-10-30

**Authors:** Adele Costabile, Sara Santarelli, Sandrine P. Claus, Jeremy Sanderson, Barry N. Hudspith, Jonathan Brostoff, Jane L. Ward, Alison Lovegrove, Peter R. Shewry, Hannah E. Jones, Andrew M. Whitley, Glenn R. Gibson

**Affiliations:** 1 Department of Food and Nutritional Sciences, The University of Reading, Reading, United Kingdom; 2 King’s College London, Biomedical & Health Sciences, Dept. of Nutrition and Dietetics, London, United Kingdom; 3 Rothamsted Research, Harpenden, Hertfordshire, United Kingdom; 4 School of Agriculture, Policy and Development, Earley Gate, Reading, United Kingdom; 5 Bread Matters Limited, Macbiehill Farmhouse, Lamancha, West Linton, Peeblesshire, Scotland; Wageningen University, Netherlands

## Abstract

A variety of foods have been implicated in symptoms of patients with Irritable Bowel Syndrome (IBS) but wheat products are most frequently cited by patients as a trigger. Our aim was to investigate the effects of breads, which were fermented for different lengths of time, on the colonic microbiota using *in vitro* batch culture experiments. A set of *in vitro* anaerobic culture systems were run over a period of 24 h using faeces from 3 different IBS donors (Rome Criteria–mainly constipated) and 3 healthy donors. Changes in gut microbiota during a time course were identified by fluorescence *in situ* hybridisation (FISH), whilst the small -molecular weight metabolomic profile was determined by NMR analysis. Gas production was separately investigated in non pH-controlled, 36 h batch culture experiments. Numbers of bifidobacteria were higher in healthy subjects compared to IBS donors. In addition, the healthy donors showed a significant increase in bifidobacteria (P<0.005) after 8 h of fermentation of a bread produced using a sourdough process (type C) compared to breads produced with commercial yeasted dough (type B) and no time fermentation (Chorleywood Breadmaking process) (type A). A significant decrease of δ-*Proteobacteria* and most *Gemmatimonadetes* species was observed after 24 h fermentation of type C bread in both IBS and healthy donors. In general, IBS donors showed higher rates of gas production compared to healthy donors. Rates of gas production for type A and conventional long fermentation (type B) breads were almost identical in IBS and healthy donors. Sourdough bread produced significantly lower cumulative gas after 15 h fermentation as compared to type A and B breads in IBS donors but not in the healthy controls. In conclusion, breads fermented by the traditional long fermentation and sourdough are less likely to lead to IBS symptoms compared to bread made using the Chorleywood Breadmaking Process.

## Introduction

Irritable bowel syndrome (IBS) is a common functional gastrointestinal disorder defined by the coexistence of abdominal discomfort or pain associated with alterations in bowel habits [1]. Several studies have indicated that the aetiology of IBS is most likely multi-factorial, due to abnormalities in intestinal motility, visceral hypersensitivity, altered brain-gut interaction, food intolerance, abnormal gut microbiota and persistence of low-grade inflammatory conditions [2]. Due to effects on modulating the immune function, motility, secretion and gut sensation, probiotics have been suggested to have the potential to exert a beneficial role in managing IBS symptoms [3]. Furthermore, it has been suggested that IBS patients could be characterized by a potential dysregulation in energy homeostasis and liver function, which may be improved through probiotic supplementation [4]. A recent review of clinical trials using lactic acid bacteria (LAB) in patients with IBS [5] showed improvement in abdominal pain, discomfort, abdominal bloating and distension as main endpoints. Dietary factors are also important in IBS as they are considered major drivers for changes in the compositional and functional relationship between microbiota and the host [6]. In fact, dietary components are substrates for metabolism by the intestinal microbial ecosystem, particularly influencing the growth and metabolic activities of dynamic bacterial populations thriving in the human colon. Studies on the relationships between diet and symptoms in IBS suggest that elimination of potential culprit foods can be helpful [7], [8]. A variety of foods are thought to contribute to IBS, but wheat is the dietary ingredient frequently cited by patients as a trigger, with the exclusion of bread and other wheat products often leading to partial or complete resolution of symptoms [7], [9]. In particular, changes in the type of bread generally available to consumers and the overall wheat content of the average diet may be significant underlying reasons why problems of gas-related gastrointestinal problems have increased. However, few studies have examined the impact of different types of bread on gastrointestinal symptoms in IBS and this is a topic worthy of further consideration [9]. To date, there is evidence that a diet low in fermentable carbohydrates, particularly fermentable oligosaccharides, disaccharides, monosaccharides and polyols (FODMAPs; also referred to as fermentable short-chain carbohydrate) reduces some symptoms associated with IBS [10]–[11]. In particular, Gibson and Shepherd suggest that fermentable short-chain carbohydrates can be a ‘problem high food source’ for those susceptible to IBS when consumed in large amounts (no specific number suggested) [10]. A recent study found that significantly more patients with IBS who followed a low-FODMAP diet (76%) reported satisfaction with their symptom response (decrease in symptoms) compared with patients following a standard diet recommended by the National Institute for Health and Clinical Excellence (54%) [11]. Although interesting, it is not possible to say which particular FODMAPs or sources of these are associated with gastrointestinal symptoms. Therefore, based on these studies, no conclusions about the impact of bread (or specific types of bread) on gastrointestinal symptoms in IBS sufferers can be drawn, although, this would seem to be a topic worthy of consideration.

A component of bread that has been suggested to help relieve IBS symptoms by shortening transit time (mainly in those suffering from constipation) is dietary fibre. However, two systematic reviews found no effect of cereal bran on IBS symptoms [12]–[13]. In fact, insoluble fibre, the main fibre component of bran, may increase symptoms in some IBS sufferers among whom reducing intakes of insoluble fibre may reduce symptoms. Therefore, eating white rather than whole meal bread may actually help relieve symptoms [14]–[15]. More specifically, a change in the process of wheat fermentation from the traditional long fermentation process to the shorter, incomplete fermentation of the Chorleywood Breadmaking Process (CBP) may have contributed to intolerance to bread through effects on gut microbiota and fermentation. Furthermore, another component of the CBP that has been suggested to be related to gastrointestinal symptoms is an increased percentage of yeast used in the fermentation process. However, whereas no evidence supporting the role of yeasts in the production of symptoms has been reported from clinical trials, dietary elimination of yeasts and anti-fungal therapy have been shown to be beneficial in IBS subjects [16]. Therefore, it is not possible to confirm or reject claims that the higher amount of yeast added to dough of bread made with CBP may be responsible for gastrointestinal problems.

Recently, there has been a growing interest in investigating the role of an altered gut microbiota in the pathogenesis of IBS [17]–[22]. “Healthy” gut microbiota have either direct bactericidal effects or can prevent the adherence of pathogenic bacteria to the wall of the gastrointestinal tract [23]. Dysbiosis in the gut may facilitate the adhesion of enteric pathogens in the human gut, which can be associated with IBS symptoms [23]. Alteration in the composition of the healthy microbiota and disturbed colonic fermentation in IBS patients may play an important role in development of IBS symptoms. Intestinal inflammation is generally believed to be associated with a reduced bacterial diversity and, in particular, a lower abundance of, and a reduced complexity in, the *Bacteroidetes* and *Firmicutes* phyla with a specific reduction of abundance in the *Clostridium* coccoides groups [24]. It has also been indicated that while *Firmicutes* are reduced there is an increase in gammaproteobacteria in patients with IBS [25]. In contrast to the general microbial dysbiosis theory, some researchers have suggested the involvement of specific taxa [26]. There have been a number of studies that have also highlighted a lower abundance of *F. prausnitzii* [26].

In the present study, we investigated the impact of breads fermented for different lengths of time on the human intestinal microbiota, using *in vitro* batch culture experiments with faecal donors from IBS patients and healthy control subjects. The main bacterial groups of the faecal microbiota were determined using 16S rRNA-based analyses. Metabolic effects of the breads on the microbial physiology were also studied using high resolution ^1^NMR-based metabolic profiling. Finally, the *in vitro* gas production was determined in non-pH-controlled, 36 h faecal static batch cultures. As such, the intention was to assess the influence of bread making process on gut microbial fermentation *in vitro*.

## Materials and Methods

### Preparation of three selected breads

Grain of wheat (cv Maris Widgeon) was milled commercially to an extraction rate of 85% and was kindly supplied by Mr Andrew Whitley and Bread Matters Limited (Macbiehill Farmhouse, Lamancha, West Linton, Peeblesshire EH46 7AZ). Three types of bread, A) conventional yeasted dough, zero bulk fermentation time; B) conventional yeasted 16-hour sponge-and-dough and C) 30% sourdough, 4-hour refreshment stage, 5-hour final proof were produced. Type A was prepared accordingly to the Chorleywood Breadmaking Process (CBP). Types B and C include the metabolism of endogenous flour components (yeasts, LAB, enzymes, micro- and macro-nutrients) that are present in greater quantity in flours containing more of the germ and bran layers.

All different type of breads (A, B and C) were prepared in the Food Processing Centre (FPC) of the Department of Food and Nutritional Sciences at the University of Reading (UK).

### Simulated human digestion of bread (from mouth to small intestine)

Frozen bread samples were thawed and 60 g of each sample was processed by an *in vitro* simulation of upper gut digestion and freeze dried as described by Maccaferri et al. [27]. Dialyses with membrane of 100–200 Daltons cut off (Spectra/por 100–200 Da MWCO dialysis membrane, Spectrum Laboratories Inc., UK) were used to remove monosaccharides from the pre-digested breads.

### Compositional analyses of the dough and breadflour samples by ^1^H NMR

NMR sample preparation was carried out according to a modification of the procedures described [28]–[29]. Extraction into 80∶20 D_2_O:CD_3_OD (1 mL) containing 0.05% w/v d_4_-TSP (1 mL) was performed for three technical replicates, of 30 mg, for each biological sample. ^1^H NMR data were collected as described below.

After analysis, to minimize variation due to differing sample pH, samples were evaporated and reconstituted in sodium phosphate buffer in D_2_O (750 µL, pH = 6, 300 mM) and data collection repeated. ^1^H^–^NMR spectra were acquired under automation at 300°K on an Avance Spectrometer (Bruker Biospin, Coventry, UK) operating at 600.0528 MHz and equipped with a 5 mm selective inverse probe. Spectra were collected using a water suppression pulse sequence with a relaxation delay of 5 s. Each spectrum was acquired using 128 scans of 64 k data points with a spectral width of 7309.99 Hz. Spectra were automatically Fourier transformed using an exponential window with a line broadening value of 0.5 Hz. Phasing and baseline correction were carried out within the instrument software (Topspin v.2.1 and Amix (Analysis of MIXtures software, v.3.9.11), Bruker Biospin). ^1^H chemical shifts were referenced to d_4_-TSP at δ0.00. Quantification of individual metabolites was achieved using Chenomx Profiler (Chenomx Inc., Alberta) software against an in-house reference library of metabolite signatures of authentic compounds, with known concentrations, ran under identical conditions.

### Collection and stool sample preparation

Faecal samples were obtained from 3 healthy human volunteers (two males, one female; age 30 to 38 years; BMI: 18.5–25) who were free of known metabolic and gastrointestinal diseases (e.g. diabetes, ulcerative colitis, Crohn’s disease, irritable bowel syndrome, peptic ulcers and cancer). All healthy faecal donors had the experimental procedure explained to them and were given the opportunity to ask questions. All donors then provided verbal informed consent for the use of their faeces in the study and a standard questionnaire to collect information regarding the health status, drugs use, clinical anamnesis, and lifestyle was administrated before the donor was ask to provide a faecal sample. The University of Reading research Ethics Committee exempted this study from review because no donors were involved in any intervention and waived the need for written consent due to the fact the samples received were not collected by means of intervention. For the IBS donors (Rome criteria - mainly constipated), written informed consent was obtained in each case and the study was approved by the St. Thomas' Hospital Research Ethics Committee (Ref 06/Q0702/74 - A study of mucosal and luminal bacteria microbiota in irritable bowel syndrome). All faecal samples collected from healthy and IBS donors were collected on site, kept in an anaerobic cabinet (10% H_2_, 10% CO_2_ and 80% N_2_) and used within a maximum of 15 minutes after collection. Samples were diluted 1/10 w/v in anaerobic PBS (0.1 mol/L phosphate buffer solution, pH 7.4) and homogenised (Stomacher 400, Seward, West Sussex, UK) for 2 minutes at 460 paddle-beats.

### *In vitro* fermentations

Sterile stirred batch culture fermentation systems (50 ml working volume) were set up and aseptically filled, with 45 ml sterile, pre-reduced, basal medium [peptone water 2 g/L (Oxoid), yeast extract 2 g/L (Oxoid, Basingstoke, UK), NaCl 0.1 g/L, K_2_HPO_4_ 0.04 g/L KH_2_PO_4_ 0.04 g/L, MgSO_4_.7H_2_O 0.01 g/L, CaCl_2_.6H_2_O 0.01 g/L, NaHCO_3_ 2 g/L, Tween 80 2 mL (BDH, Poole, UK), haemin 0.05 g/L, vitamin K1 10 µL, cysteine.HCl 0.5 g/L, bile salts 0.5 g/L, pH 7.0)] and gassed overnight with oxygen free nitrogen (15 mL/min). The different pre-digested breads, 5 g (1/10 w/v) were added to the respective fermentation vessels just prior to the addition of the faecal slurry. The temperature was kept at 37°C and pH was controlled between 6.7 and 6.9 using an automated pH controller (Fermac 260, Electrolab, Tewkesbury, UK). Each vessel was inoculated with 5 ml of fresh faecal slurry (1/10 w/w) for both healthy and IBS donors. The batch cultures (n = 3) were ran over a period of 24 h and 5 mL samples were obtained from each vessel at 0, 4, 8 and 24 h for fluorescence *in situ* hybridisation (FISH) and ^1^H NMR analysis.

### *In vitro* enumeration of bacteria population by FISH

Numbers of predominant intestinal bacterial groups, as well as total bacterial populations, were evaluated in samples from *in vitro* batch culture system by fluorescence *in situ* hybridization (FISH) analysis, as previously described by Martin-Pelaez and colleagues [30]. The probes used are reported in Table 1. They were commercially synthesised and 5′-labelled with the fluorescent Cy3 dye (Sigma, UK).

**Table 1:** Oligonucleotide probes used in this study for FISH analysis.

Probe	Target group	Reference
EUB338§	Most bacteria	[15]
EUB338II§	Most bacteria	[15]
EUB338III§	Most bacteria	[16]
Bac303	*Bacteroides* spp.	[17]
Bif164	*Bifidobacterium* spp.	[17]
Lab158	*Lactobacillus-Enterococcus* spp.	[18]
Erec482	Most of the *Clostridium* *coccoides*-*Eubacterium rectale* group(*Clostridium* cluster XIVa and XIVb)	[19]
Chis150	*Clostridium histolyticum* group	[19]
Prop853	*Clostridium* cluster IX	[20]
Delta496a-b-c	*Deltaproteobacteria*-*Gemmatimonadetes* group	[21]

§ These probes are used together in equimolar concentrations.

### Short chain fatty acid analysis

Analysis was performed using ion exclusion high performance liquid chromatography (HPLC) system (LaChrom Merck Hitachi, Poole, Dorset UK) equipped with pump (L-7100), RI detector (L-7490) and autosampler (L-7200). Samples (1 mL) from each fermentation time point (1 mL) were centrifuged at 13,000×g for 10 min to remove bacterial cells and any particulate material. Supernatants were filtered through a 0.22 µm filter unit (Millipore, Cork, Ireland) and 20 µL injected into the HPLC, operating at a flow rate of 0.5 mL/min with heated column at 84.2°C.SCFAs (acetate, propionate, butyrate) and lactate were determined by HPLC on an Aminex HPX-87H column (300×7.8 mm, Bio-Rad, Watford, Herts, UK). Degassed 5 mM H2SO4 was used as eluent at a flow rate of 0.6 ml/min and an operating temperature of 50°C. Organic acids were detected by UV at a wavelength of 220 nm, and calibrated against standards of corresponding organic acids at concentrations of 12.5, 25, 50, 75 and 100 mM. Internal standard of 20 mM 2-ethylbutyric acid was included in the samples and external standards.

### 1H NMR Metabolomic profile of supernatants from fermentation

The fermentation supernatant from all time points was freeze-dried, dissolved in 600 µL of phosphate buffer 0.2 M (pH 7.4) in D_2_O plus 0.001% TSP and 550 µL transferred into 5 mm NMR tubes for analysis. All NMR spectra were acquired on a Bruker Avance DRX 700 MHz NMR Spectrometer (Bruker Biopsin, Rheinstetten, Germany) operating at 700.19 MHz and equipped with a CryoProbe from the same manufacturer [28]–[29]. They were acquired using a standard 1-dimensional (1D) pulse sequence [recycle delay (RD)-90°-t1-90°-tm-90°-acquire free induction decay (FID)] with water suppression applied during RD of 2 s, a mixing time (tm) of 100 ms and a 90° pulse set at 7.70 µs. For each spectrum, a total of 128 scans were accumulated into 64 k data points with a spectral width of 14005 Hz. A range of 2D NMR spectra were performed on the same equipment for selective samples, including correlation spectroscopy (COSY), total correlation spectroscopy (TOCSY) and heteronuclear single quantum coherence (HSQC) NMR spectroscopy. The FIDs were multiplied by an exponential function corresponding to 0.3 Hz line broadening. All spectra were manually phased, baseline corrected and calibrated to the chemical shift of TSP (δ 0.00). Metabolites were assigned using our in house standard database, data from literature [37]–[38] and confirmed by 2D NMR experiments.

### Gas production rate determinations

Sterile glass tubes (18×150 mm, Bellco, Vineland, New Jersey, USA) containing 13.5 mL pre-reduced basal medium [peptone water 2 g/L (Oxoid), yeast extract 2 g/L (Oxoid, Basingstoke, UK), NaCl 0.1 g/L, K_2_HPO_4_ 0.04 g/L, MgSO_4_.7H_2_O 0.01 g/L, CaCl_2_.6H_2_O 0.01 g/L, NaHCO3 2 g/L, Tween 80 2 mL (BDH, Poole, UK), haemin 0.05 g/L, vitamin K1 10 µL, cysteine-HCl 0.5 g/L, bile salts 0.5 g/L, pH 7.0)] were placed into the anaerobic cabinet and kept overnight. Pre-digested breads (1% w/v) were added to the fermentation tubes just prior to addition of the faecal inocula (1/10 w/v) [39]. The tubes were then sealed with a gas impermeable butyl rubber septum (Bellco, Vineland, New Jersey, USA) and aluminium crimp (Sigma Aldrich, Gillingham, Dorset, UK). Gas production was evaluated by recording the headspace pressure (pounds per square inch; psi) from each vial. Gas production experiments were performed in four replicates for each type of bread. Vials were incubated at 37°C and continuously shaken. Pressure readings were obtained every 3 h up to 36 h fermentation period by piercing the rubber caps with a U200/66 needle adaptor connected to a pressure transducer (type 2200BGF150WD3DA; Keller Ltd, Dorchester, Dorset, UK) with a T443A digital panel meter (Bailey and Makey Ltd, Birmingham, UK). Pressure readings (psi) were converted into gas volume (mL) using an established linear regression of pressure recorded in the same vials with known air volumes at the incubation temperature.

### Statistical analysis

Differences between bacterial counts and SCFA profiles at 0, 4, 8 and 24 h fermentation for each substrate were tested for significance using paired t-tests assuming equal variances and considering a two-tailed distribution. To determine whether there were any significant differences in the effect of the substrates; differences at each time point were tested using 2-way ANOVA with Bonferroni post-test. All analyses were performed using GraphPad Prism 5.0 (GraphPad Software, La Jolla, CA, USA).

Metabolic profiles of fermentation supernatant were imported into Matlab version R2010b (Mathworks UK) and statistical algorithms were provided by Korrigan Sciences (Korrigan Sciences Ltd, UK). To minimise variability due to water pre-saturation, the water resonance region (δ 4.70–5.05) was removed. Data were then normalised to the probabilistic quotient as previously described [40]. All statistical models were performed using unit variance scaling. Principal component analyses (PCA) were performed on all spectra in order to detect any outliers and to identify patterns associated with volunteers, time, fermentation condition or donor group. In order to optimise statistical separation between samples derived from IBS and control donors at 24 h, a partial least square discriminant analysis was also performed using one predictive component. This later model was validated using 1000 random permutations, and a p value was calculated by rank determination of the model actual Q^2^Y value (representing the goodness of prediction) among the Q^2^Y values calculated for the permutated models. Finally, in order to focus on the ethanol production in control- and IBS-derived samples, the area under the ethanol triplet at 1.18 ppm was integrated and an ANOVA followed by a multiple comparison test (TukeyHSD) were performed in R (version 2.15).

## Results

### Compositions and properties of flour, doughs and breads

A sample of wheat cv Maris Widgeon was milled to 85% extraction rate to give a flour fraction enriched in fibre and other components derived from the bran and germ, compared to pure white flour which is derived solely from the starchy endosperm. This flour was similar to those used by many artisan bakers in the UK. Three types of bread were produced, with yeast but zero bulk fermentation (similar to the Chorleywood Breadmaking Process (CBP) (which is used widely for factory production of bread in the UK and many other countries) (type A), with yeast and 16 hours fermentation (type B) and a sourdough process using a “starter dough” and a total of 9 hours fermentation (type C). The composition of major soluble polar metabolites in the flour, doughs and bread samples were determined by ^1^H^–^NMR (Table 2). Typically, flour contained lower amounts of the abundant free sugars (maltose, glucose and fructose) that tended to be broken down in the dough samples by a longer fermentation process. Less abundant flour carbohydrates included sucrose and raffinose. Sucrose levels were markedly lower in the CBP dough and bread (type A) and in the dough and bread undergoing longer fermentation (type B) but remained stable in the sourdough samples. Sugars such as arabinose, xylose and galactose were not detected in the flour spectra but were present in all the bread and dough spectra. Increasing the fermentation time did not change the amounts of these carbohydrates (CBP vs dough/bread B). However, significantly higher levels of these sugars were released by the sourdough process. In addition, the dough and bread samples produced using the sourdough process contained higher levels of glycerol and mannitol, the latter not being present in bread B or that produced using the CBP. Organic acids showed striking differences between the samples. As expected, lactate levels were increased with longer fermentation and were very high in both the sourdough (405 µmoles/g) and the sourdough bread (111.4 405 µmoles/g). Other organic acid levels such as citrate and malate also discriminated the samples. While the malate levels fell to around 30% in the long fermentation samples compared with the CBP process, the citrate levels remained stable even with a longer fermentation process but were completely absent from the sourdough product spectra, Succinate generally showed an opposite profile, increasing during longer fermentation (B) but decreasing in the sourdough products. Interestingly, dough B was the only sample containing low levels of acetic acid. In general, the sourdough dough and resultant bread had significantly higher contents of many polar metabolites than the CBP and long fermentation doughs and breads. The majority of amino acid (alanine, valine, leucine, isoleucine, glutamate, glutamine, aspartate, phenylalanine, tryptophan, tyrosine and gamma-amino butyric acid (GABA, a non-protein amino acid) were present in higher concentrations in the sourdough samples and in some cases also the bread made using this process. Similarly, signals corresponding to methionine, whose levels were not detected in other flour, bread or dough samples, were clearly present in the spectra from the sourdough samples. Notable exceptions were asparagine, which showed significantly lower levels in dough and bread B but whose levels were unchanged in the sourdough samples and threonine whose levels were decreased with a longer fermentation process and which disappeared completely in the sourdough products. Choline and glycine-betaine, which are methyl donors, were elevated in both sourdough samples and those arising from process B compared to the CBP products. Ethanol, a product of the breadmaking process was present in all dough and bread samples and was typically higher in samples receiving a longer fermentation. All doughs had lower levels of raffinose and maltose than their corresponding bread products, which is consistent with their use as substrates during proofing.

**Table 2:** Quantification (µmoL/g dry wt) of selected metabolites in flour, dough A, B and C and breads A, B and C.

	Flour	Dough A (CBP)	Dough B	Dough C	Bread A (CBP)	Bread B	Bread C
Carbohydrates							
Glucose	2.693±0.577	34.072±1.185	22.133±1.702	129.00±11.68	30.873±9.117	13.339±1.297	45.036±1.062
Fructose	4.587±1.844	45.220±2.219	29.081±1.942	20.456±8.571	44.756±3.048	24.417±5.238	8.601±5.868
Maltose	7.660±1.007	41.521±3.981	27.471±1.014	31.160±2.643	101.420±0.171	53.900±5.684	78.210±9.105
Galactose	n.d.	1.168±0.345	1.196±0.544	9.506±1.017	0.814±0.234	0.799±0.660	1.911±0.723
Sucrose	2.199±0.302	0.414±0.073	0.281±0.052	1.912±0.256	0.421±0.087	0.441±0.087	2.413±0.072
Raffinose	2.194±0.245	2.176±1.548	1.129±0.824	2.754±1.312	2.487±0.363	1.607±0.396	5.407±0.386
Xylose	n.d.	0.992±0.596	1.324±0.640	11.280±3.900	1.009±0.432	0.879±0.408	2.443±0.569
Trehalose	n.d.	n.d.	n.d.	9.147±0.943	1.706±0.329	0.713±0.078	n.d.
Arabinose	n.d.	0.916±0.230	2.523±0.347	19.519±2.609	1.221±0.282	2.652±1.717	2.153±0.053
Sugar alcohols							
Glycerol	4.111±0.594	19.148±5.771	23.648±8.953	32.329±3.190	17.733±5.763	31.106±3.708	13.986±1.875
Mannitol	n.d.	5.944±5.421	7.488±1.305	86.124±7.017	n.d.	n.d.	35.170±0.751
Organic Acids							
3-Hydroxyisobutyrate	n.d.	0.167±0.012	n.d.	n.d.	0.207±0.003	n.d.	n.d.
Citrate	0.912±0.102	0.961±0.041	1.019±0.099	n.d.	1.472±0.187	0.876±0.363	n.d.
Fumarate	0.453±0.012	0.626±0.071	0.304±0.063	0.092±0.019	0.647±0.050	0.503±0.038	0.637±0.118
Malate	5.664±0.168	5.942±0.390	1.692±0.169	3.054±0.907	7.060±0.497	1.894±0.426	n.d.
Succinate	0.427±0.007	1.111±0.072	1.612±0.098	1.032±0.238	1.317±0.087	1.572±0.133	0.873±0.035
Formate	0.352±0.011	0.490±0.019	0.487±0.093	0.762±0.059	0.586±0.109	0.934±0.127	0.710±0.098
Lactate	0.857±0.057	1.058±0.199	11.638±0.624	404.89±46.022	1.048±0.166	11.233±1.206	111.417±3.443
Amino Acids							
Alanine	0.526±0.025	0.989±0.076	1.041±0.054	5.284±0.396	1.146±0.062	0.961±0.125	1.622±0.066
Asparagine	1.543±0.055	1.613±0.087	0.494±0.045	2.039±0.699	1.719±0.018	0.724±0.254	2.191±0.202
Aspartate	1.837±0.070	1.736±0.117	1.341±0.063	9.310±1.008	2.017±0.111	1.272±0.185	3.531±0.080
GABA	0.249±0.072	0.796±0.050	1.031±0.080	5.699±0.561	0.917±0.089	0.939±0.167	2.199±0.075
Glutamate	1.203±0.369	1.129±0.532	0.546±0.065	8.321±2.195	0.974±0.114	0.651±0.231	1.587±0.321
Glutamine	0.558±0.047	0.726±0.097	0.448±0.142	3.371±0.239	0.601±0.106	0.367±0.047	0.783±0.058
Leucine	0.139±0.045	0.250±0.075	0.221±0.057	14.050±1.599	0.342±0.091	0.149±0.021	2.246±0.733
Isoleucine	0.129±0.039	0.159±0.036	0.158±0.063	4.113±0.234	0.191±0.025	0.118±0.030	0.666±0.047
Methionine	n.d.	n.d.	n.d.	2.436±0.248	n.d.	n.d.	0.323±0.009
Phenylalanine	n.d.	0.178±0.022	0.124±0.069	4.912±0.446	0.186±0.050	n.d.	0.853±0.078
Threonine	0.132±0.047	0.487±0.166	0.378±0.098	n.d.	0.347±0.018	n.d.	n.d.
Tryptophan	0.800±0.056	0.782±0.014	0.509±0.132	2.570±0.327	0.921±0.071	0.473±0.094	1.281±0.042
Tyrosine	0.132±0.022	0.167±0.022	0.171±0.041	2.762±0.451	0.192±0.034	0.152±0.044	0.497±0.044
Valine	0.173±0.032	0.284±0.034	0.270±0.036	6.993±0.219	0.384±0.017	0.230±0.033	1.344±0.035
Methyl Donors							
Betaine	8.464±0.658	8.919±1.826	9.903±0.458	22.930±1.860	6.262±0.289	8.070±4.125	15.688±0.401
Choline	1.103±0.066	1.798±0.092	2.139±0.076	3.266±0.239	1.570±0.098	1.917±0.185	2.508±0.060
Choline-O-Sulfate	0.301±0.044	0.530±0.038	0.613±0.054	2.941±0.297	0.424±0.049	0.519±0.040	0.908±0.005
Acetylcholine	0.048±0.005	0.042±0.002	0.042±0.008	1.999±0.092	0.049±0.005	0.043±0.010	0.358±0.008
Phosphocholine	0.062±0.007	0.053±0.003	0.060±0.006	2.593±0.120	0.204±0.250	0.056±0.015	0.463±0.009
Trigonelline	0.076±0.008	0.089±0.015	n.d.	n.d.	0.083±0.020	n.d.	n.d.
Miscellaneous							
Ethanol*	0.907±0.098	14.802±0.437	21.350±0.839	30.112±4.874	29.49±0.384	37.918±9.78	17.340±0.325
Putrescine	n.d.	n.d.	n.d.	n.d.	n.d.	n.d.	1.074±0.175
Adenine	n.d.	n.d.	n.d.	0.663±0.173	n.d.	n.d.	0.636±0.039
Adenosine	0.070±0.012	0.222±0.012	0.090±0.003	0.093±0.015	0.343±0.109	0.179±0.013	0.063±0.014
Uridine	0.109±0.027	0.263±0.057	0.156±0.010	n.d.	0.339±0.078	0.207±0.052	n.d.

n.d. denotes metabolite not detected at above 0.075 micromoles/g.

Errors are standard deviations of 3 replicates.

* denotes data obtained from an 80∶20 D_2_O:CD_3_OD extraction rather than buffer at pH 6.5.

### Changes in faecal microbiota measured by FISH

Eight 16S rRNA-based fluorescence *in situ* hybridisation (FISH) probes were used to identify predominant groups, or species, of human faecal microbiota before and after incubation with digested bread samples (Table 1). Bacterial numbers of the samples from IBS donors were compared to the samples obtained from healthy subjects (Table 3). Numbers of bifidobacteria were higher in the control group compared to the IBS donors. A significant increase in bifidobacterial populations occurred (P<0.005) after 8 hours of fermentation in bread produced with sourdough (type C) for healthy donors compared to breads produced with commercial yeast dough and no time fermentation (type A). No significant changes were also noted in *Bacteroides-Prevotella* group populations (detected by Bac303) at all time points in IBS donors. However, all type of breads stimulated the growth of bacteria detected by Bac303 at 8 h and over 24 hours fermentation in healthy donors, but there was no significant difference compared to the control substrate. No significant differences were detected for *Clostridium histolyticum* subgroup (detected by Chis150) and lactobacilli in IBS and healthy donors. Significant decreases in δ-Proteobacteria and most *Gemmatimonadetes* (enumerated by probe DELTA495 a-b-c), which are sulphate-reducing microorganisms, was observed after 24 h fermentation of type C bread in IBS and healthy donors. This may be due to the ability of the sourdough bread to enhance the growth of beneficial bacteria rather than undesirable microorganisms. Cluster IX representatives (detected by Prop853) were increased by bread type C at 8 h and 24 h in both donor types.

**Table 3:** Bacterial populations (Mean value Log_10_ cells mL± SD) in pH controlled and stirred batch.

Probe	Time (h)	Negativecontrol^h^	NegativeControl^IBS^	TypeA^h^	TypeA^IBS^	TypeB^h^	TypeB^IBS^	TypeC^h^	TypeC^IBS^
Bif164	0	8.22±0.13	7.75±0.35	8.26±0.16	7.58±0.20	8.44±0.25	7.63±0.07*	8.48±0.11	7.94±0.06*
	4	8.34±0.13	7.81±0.33	8.63±0.24	7.78±0.47	8.46±0.23	7.98±0.36	8.60±0.08	8.22±0.09*
	8	8.44±0.17	7.95±0.31	8.69±0.32	8.33±0.29	8.71±0.38	8.28±0.26	8.86±0.15^A^	8.29±0.28
	24	8.15±0.10	7.82±0.22	8.31±0.36^B^	7.97±0.30	8.39±0.31	7.97±0.41	8.45±0.48	8.02±0.31
Lab158	0	7.92±0.15	8.04±0.51	8.12±0.26	8.00±0.40	8.14±0.17	7.83±0.40	8.09±0.01	7.89±0.40
	4	7.68±0.14	7.82±0.23	7.99±0.14	7.70±0.13	8.07±0.20	7.73±0.49	8.00±0.19	7.76±0.17*
	8	7.82±0.36	7.85±0.21	8.34±0.32	8.04±0.32	8.21±0.23	8.06±0.29	8.16±0.72	8.05±0.38
	24	7.99±0.30	7.82±0.18	8.22±0.24	7.76±0.20**	8.11±0.07	7.78±0.32	8.20±0.23	7.84±0.08
Eub338	0	9.06±0.20	8.82±0.12	8.91±0.11	8.75±0.28	8.81±0.22	9.06±0.29	9.09±0.25	8.95±0.35
	4	8.79±0.14	9.11±0.24	9.12±0.24	9.08±0.18	8.92±0.16	8.92±0.37	8.76±0.17	9.01±0.69
	8	9.11±0.39	9.04±0.23	9.48±0.08^A^	9.41±0.26^A^	9.46±0.28^A^	9.24±0.21	9.22±0.62	9.25±0.21
	24	9.44±0.43	9.22±0.19^A^	9.45±0.21	9.50±0.18^AB^	9.76±0.19^AC^	9.40±0.40^A^	9.48±0.36	9.53±0.17^C^
Erec482	0	8.26±0.18	8.60±0.55	8.18±0.60	8.37±0.21	8.52±0.56	8.22±0.21	8.05±0.13	8.08±0.17
	4	8.29±0.30	8.26±0.43	8.36±0.13	8.25±0.19	8.33±0.48	8.12±0.56	8.37±0.35	7.93±0.18
	8	8.14±0.53	8.26±0.36	9.07±0.04^B^	8.17±0.21*	8.63±0.62	7.91±0.86	8.63±0.42	7.61±0.51
	24	8.43±0.36	8.60±0.10	8.96±0.32	8.29±0.14*	8.41±0.20	8.16±0.44	8.93±0.30	7.63±0.39
Prop853	0	7.19±0.30	8.06±0.38	7.37±0.44	8.15±0.35	7.53±0.44	8.19±0.39	7.50±0.39	7.74±0.20
	4	7.76±0.40	8.27±0.34	8.19±033	8.02±0.17	7.98±0.28	7.55±0.25	8.23±0.24	7.48±0.17^A^ *
	8	8.17±0.01^A^	8.23±0.10	8.52±0.17	8,13±0.20	8.45±0.34^B^	8.03±0.23	8.21±0.31	8.09±0.23^AB^
	24	8.04±0.20^A^	8.47±0.08^C^	8.19±0.38	8.14±0.21	8.00±0.22	8.13±0.04	8.27±0.21^A^	8.12±0.18^AB^
Bac303	0	8.16±0.17	8.20±0.14	8.19±0.35	8.35±0.07	8.13±0.06	8.35±0.23	7.79±0.15	8.26±0.24
	4	7.98±0.47	8.46±0.22	8.50±0.14	8.30±0.29	8.14±0.40	8.45±0.11	8.16±0.51	8.43±0.33
	8	8.22±0.20	8.28±0.18	8.79±0.29	8.23±0.17	8.82±0.48^B^	8.50±0.21	9.22±0.62^A^	8.46±0.10
	24	8.43±0.34	8.29±0.02	9.20±0.15^AB^	8.35±0.35*	8.61±0.59	8.29±0.20	8.84±0.26^A^	8.48±0.24
Chis150	0	7.68±0.61	8.12±0.42	7.88±0.56	8.05±0.49	7.86±0.49	8.07±0.51	8.17±0.03	8.31±0.45
	4	8.18±0.27	8.31±0.39	8.10±0.06	8.02±0.51	7.88±0.28	7.98±0.45	8.04±0.39	8.22±0.09
	8	8.11±0.70	8.26±0.29	8.30±0.50	8.09±0.28	8.27±0.71	8.39±0.14	8.41±0.44	8.36±0.21
	24	7.93±0.68	8.14±0.08	8.08±0.67	8.34±0.36	8.14±0.62	8.07±0.40	8.26±0.64	7.82±0.68
Delta496a-b-c	0	7.43±0.50	6.95±0.13	7.31±0.51	7.12±0.13	7.36±0.32	7.11±0.27	7.30±0.58	6.89±0.49*
	4	7.67±0.15	7.09±0.09*	7.52±0.16	7.80±0.13^A^	7.38±0.30	7.93±0.24*	7.49±0.24	7.48±0.28
	8	7.63±0.15	7.52±0.50	7.52±0.35	8.08±0.10^A^	7.49±0.31	8.03±0.15	7.55±0.30	7.28±0.25
	24	7.50±0.58	7.18±0.34	7.38±0.28	7.83±0.28^A^	7.20±0.07	8.05±0.33^A^	7.16±0.23^C^	7.45±0.24

cultures at 0, 4, 8 and 24 inoculated with healthy^h^ and IBS faecal microbiota^IBS.^

A Significantly different from 0 h for the same substrate.

B significantly different from 4 h for the same substrate.

C significantly different from 8 h for the same substrate (paired t-test, p<0.05).

* p<0.05, ** p<0.01, *** p<0.001 Significantly different from control (without any additional substrate) using two-way ANOVA with Bonferroni post-test.

### Short chain fatty acid analysis

Short chain fatty acids (SCFAs), which are the principal end products of gut bacterial metabolism, were measured after 0, 4, 8 and 24 h fermentation with the different test substrates using HPLC analysis. All substrates gave significant increases in total SCFA concentration after 8 h of fermentation in both donor types with fermentation of type C bread leading to significant increases in concentrations of butyrate after 8 h fermentation in both donor groups. Acetate was the dominant SCFA produced after 24 h fermentation with all breads and in both IBS and healthy donors. Data are shown in Table 4.

**Table 4:** Short chain fatty acids production ± SD by bread fermentations in pH controlled and stirred batch cultures at 0, 4, 8 and 24 inoculated with healthy^h^ and IBS faecal microbiota^IBS^.

	Time (h)	Negative control^h^	Negative control^IBS^	Type A^h^	Type A^IBS^	Type B^h^	Type B^IBS^	Type C^h^	TypeC^IBS^
**Total**	0	0±0.00	0±0.00	0.22±0.38	0.15±0.26	0±0.00	0.26±0.40*	0±0.00	0.68±0.19*
	4	8.20±10.5	12.26±17.0	6.30±6.10	7.01±6.20	1.30±1.75	0.00±0.36	0.00±0.08	1.90±1.85*
**production**	8	8.44±0.17	7.95±0.31	8.69±0.32	8.33±0.29	8.71±0.38	8.28±0.26	8.86±0.15a	8.29±0.28
	24	6.50±1.63	7.01±1.63	50.83a±12.3***	43.0a ±14.0***	49.15a ±15.6***	51.98a ±16.0***	52.00 a ±12.0***	59.0 a ±5.0 ***
	0	0.00±0	0.22±0.38	0.16±0.26	0.00±0.00	0.26±0.00	0.53±0.20	0.19±0.01	0.05±0.30
**Acetic acid**	4	1.40±1.22	1.33±1.22	7.20±7.14	7.70±0.13	0.07±0.20	7.73±0.49	4.10±3.19	9.67±9.16***
	8	1.30±0.36	1.30±0.30	5.34±8.32	25.01 a ±15.12**	18.21±7.23	8.06±0.29	12.65 a,b±17.72	27.38 a,b ±14.19
	24	3.99±0.30	5.82±0.18	40.22 a,c±8.24***	47.76 a,c±5.20***	36.41a,b ±7.07***	7.78±0.32	32.20 a,b,c ±12.23	34.85 a,b,c ±9.53***
	0	0.05±0.08	0.00±0.12	0.91±0.11	0.75±0.28	0.00±0.22	0.06±0.29	0.09±0.25	0.95±0.35
**Propionic acid**	4	0.50±0.61	1.11±3.12	3.26±3.88	7.26±7.88	0.92±0.16	1.26±1.88	1.59±2.76	1.26±1.88
	8	0.42±0.12	0.60±1.05	4.88±6.48	8.88±10.48	5.88±10.2	2.88±4.48	0.22±0.00	2.88±4.48
	24	1.70±0.59	5.22±3.19	9.45±0.21	14.54±13.56	7.21±7.05	8.54±7.56	14.48±12.7 a	18.54±17.56 a
	0	0.05±0.08	0.11±0.16	0.00±0.02	0.00±0.00	0.00±0.20	0.00±0.01	0.05±0.13	0.08±0.17
**Butyric acid**	4	0.50±0.61	0.00±0.00	8.36±0.13	0.25±0.19	0.33±0.48	2.12±0.56	0.37±0.35	0.93±0.18
	8	0.42±0.11	0.26±0.36	11.07±8.04b	18.17±0.21*	21.63±11.62	7.91±5.86	18.63±0.42	17.61±0.51
	24	1.61±0.59	0.34±0.31	18.96±11.32	28.29±0.14*	38.41±20.20	18.16±12.44	28.93±0.30 a,b,c	32.63±0.39***

Values are mmol/L concentrations in batch culture at 0, 4, 8 and 24 h fermentation as means of three experiments with different faecal donors.

a Significantly different from initial concentration (P<0.05).

b significantly different from 4 h concentration (P<0.05).

c significantly different from 24 h concentration (P<0.05) *P<0.05, **P<0.01, ***P<0.001.

Significantly different from control (cellulose) using 2-way ANOVA with Bonferroni post-test.

### Metabolic profiling

**Figure 1:**
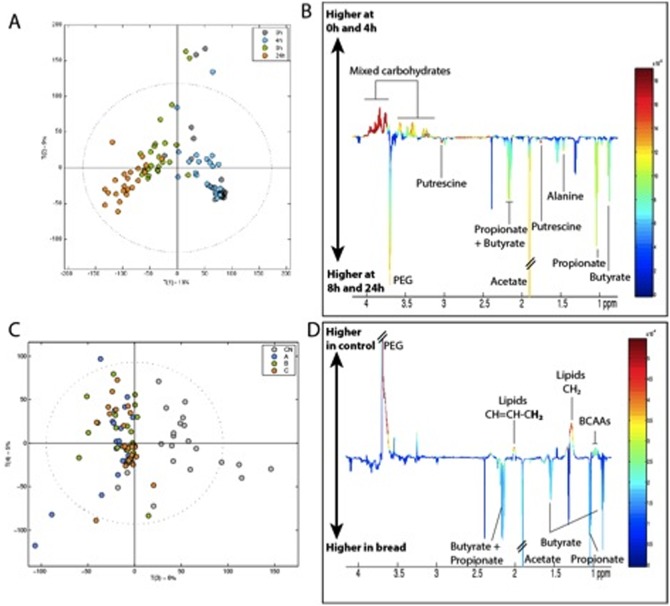
Metabolic trajectories of bread fermentated by gut bacteria obtained from both control and IBS patients (n = 3). PC1 versus PC2 scores plot (A) and PC1 loadings (B) derived from the 700 MHz ^1^H NMR spectra of fermentation supernatants color coded for collection time-points. Key: Grey: 0 h, Blue: 4 h, Green: 8 h, Orange: 24 h. PC3 versus PC4 scores plot color coded for bread (C) and PC3 loadings (D). Key: Grey: control, Blue: bread A, Green: bread B, Orange: Bread C.

Metabolic profiles of fermentation supernatants (Type A, B and C breads) were acquired at 0, 4, 8 and 24 h post inoculation by High Resolution 700 MHz NMR spectroscopy. Principal component analysis (PCA) revealed a clear trajectory over time, mainly due to decreasing carbohydrate concentration and increased production of SCFAs (Figure 1). The cluster of samples isolated by PC2 displayed a higher polar lipid content (corresponding to medium chain fatty acids).

**Figure 2:**
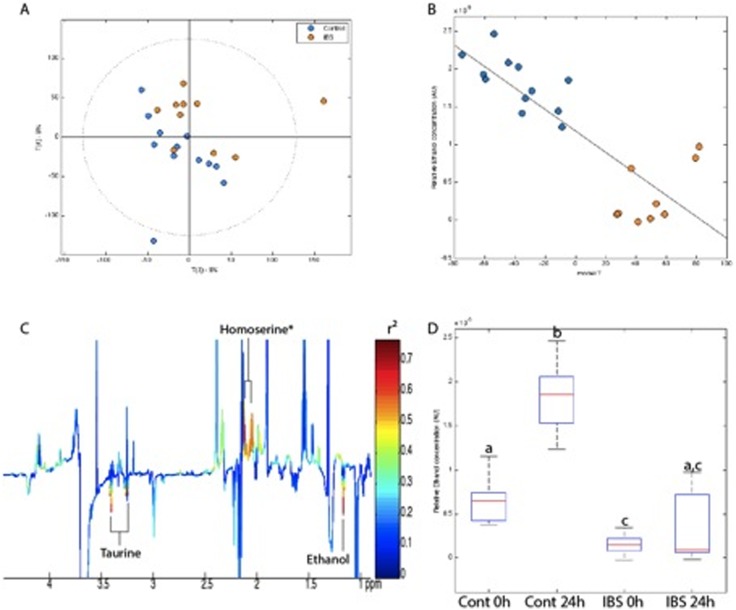
Divergent fermentation of bread samples by IBS and control microbiota. PCA scores plot (A) and O-PLS-DA scores (B) and associated loadings (C) derived from 700 MHz ^1^H NMR spectra of fermentation supernatants. Relative ethanol production derived from the integrated area under the curve of original NMR spectra for the methyl protons at 1.18 ppm. ANOVA: p<2.10^−16^; Multiple comparison test: (a) p<0.0001 different from b and c, (b) p<0.0001 different from a and c. Key: Blue: control; Orange: IBS. *putative assignement.

Supernatants from the different breads could not be statistically differentiated from one another but were all distinguished from the controls due to lower levels of polyethylene glycol (PEG), lipids and branched chain amino acids in the fermented bread samples (Figure 2). As expected, all supernatants incubated with bread samples displayed higher levels of SCFAs compared to controls (Figure 2).

While the fermentation supernatants (all types of bread) derived from the IBS and control patients could not be separated at 0 h post-fermentation, they were clearly separated at 24 h, as indicated by the PCA displayed in Figure 2A. An Orthogonal Partial Least Square (O-PLS) analysis also provided significant discrimination (R^2^Y = 0.82, R^2^X = 0.10, Q^2^Y = 0.40; permutation test based on 1000 random permutations resulted in a p value of 0.003) (Figure 2B). This separation was due to a higher content of ethanol and taurine in the controls and of proline in the IBS samples. In order to determine more precisely the extent of ethanol production after 24 h of fermentation in these 2 groups, the area under the ethanol resonance of the methyl protons at 1.18 ppm was integrated at 0 h and 24 h (Figure 2C). This shows a 4 times increase in ethanol production in control-derived samples while almost no increase was observed in IBS-derived samples. The large standard error observed in IBS-derived samples at 24 h was due to the fact that the supernatants from only one donor contained ethanol.

### Gas production kinetics

**Figure 3:**
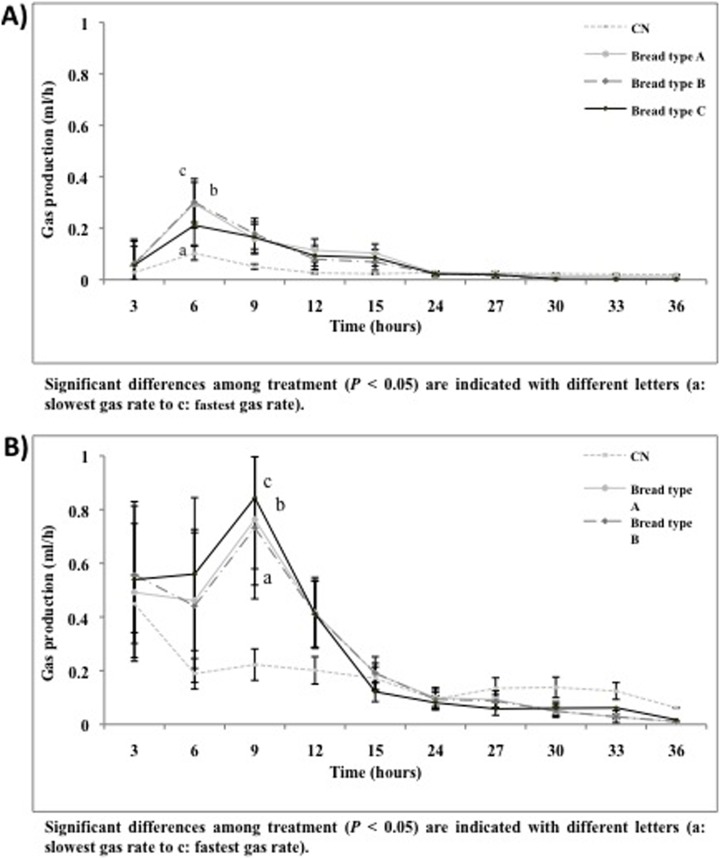
Gas production pattern expressed in mL per hour from non-pH controlled batch culture (average results ± standard deviation of 3 volunteers, *n* = 3) inoculated with healthy faecal microbiota (A); Gas production pattern expressed in mL per hour from non-pH controlled batch culture (average results ± standard deviation of 3 volunteers, *n* = 3) inoculated with IBS faecal microbiota (B).

Gas production during the 36 h of non pH-controlled faecal batch culture is shown in Figure 3. The rates of gas production for type A and B breads were almost identical in IBS and healthy donors, peaking after 6 h, and continuing for up to 36 h (Figure 3 A, B). Type C bread resulted significantly in lower rates combined with lower total gas production (data not shown) compared to the control (P<0.05). This indicates that type C was fermented more slowly to produce a more gradual build-up of gas compared to other selected breads (Figure 3, B).

## Discussion

Irritable bowel syndrome (IBS) is a common functional bowel disorder, with an estimated worldwide prevalence of 10%–20% among adults and adolescents. IBS is characterised by pain or discomfort, disturbed bowel habits and altered stool characteristics. The exact aetiology of IBS is likely to be multifactorial; moreover, patients diagnosed with the disorder may also be experiencing bowel symptoms due to different causes. Much attention has recently been focused on the impact of gastrointestinal microbiota on this disorder [20]–[26]. Indeed, in recent years, there has been much greater recognition that bloating results mainly from abnormal levels of gut fermentation. It is not known exactly which microbial agents contribute to excessive fermentation but there is evidence to support a role for both bacteria and yeasts [16]. To date, wheat is frequently cited by patients as a trigger with exclusion of bread and other wheat products often leading to partial or complete resolution of symptoms [41]–[43]. Very few studies have investigated the effects of different bread-making processes on bloating or gastrointestinal symptoms. More specifically, a change in bread making processes from a traditional long fermentation process to a short, incomplete fermentation may have contributed to bread intolerance through its effects on fermentation in the colon. However, hitherto, there is no published evidence to support claims that bread made with the Chorleywood Bread Process (CBP) affects the gastrointestinal system in a different way compared with the more traditional Bulk Fermentation Process (BFP) or other commonly used bread-making processes [10]–[12].

Our hypothesis is that bread fermented by a traditional long fermentation technique is less likely to lead to IBS symptoms, especially gas and bloating, compared to bread made using the widely used short CBP. In this context, the overall aim of this study was to compare the fermentation properties of three breads prepared with different conditions using *in vitro* batch culture. Analysis of dough and breads showed clear effects of the production process on the concentrations of polar metabolites, including carbohydrates which could affect the pattern of fermentation in the colon. For example, the sourdough process resulted in high levels of xylose, arabinose, galactose and mannitol, none of which are normally detected in flour samples. However, these differences may well be modulated by digestion and absorption in the upper part of the GI tract, and may not therefore represent the composition of the samples entering the colon.

*In vitro* studies of digested bread samples were therefore carried out to determine the impact of the processing system on the intestinal microbiota and to compare their ability to enhance faecal bifidobacteria. Bifidobacteria are of particular interest because this genus is used as a probiotic, does not produce gas, and has been tested for positive effects on IBS [5]. As expected, numbers of bifidobacteria were higher in healthy people compared to IBS donors. The increase in bifidobacteria population was also significantly higher (P<0.005) after 8 hours of fermentation of bread produced using a sourdough process (type C) for healthy people compared to breads produced with commercial yeasted dough and no time fermentation. In particular, the CBP (type A) bread showed significant increase in the bifidobacteria populations (enumerated by probe Bif164) after 24 h. No significant change was recorded in bifidobacteria numbers in IBS patients.

Short chain fatty acids (SCFAs) were determined after 0, 4, 8 and 24 h fermentation with the different test substrates via HPLC and NMR techniques. All substrates gave significant increases in total SCFAs concentrations after 24 h fermentation in both donor types. Acetate was the dominant SCFA produced in all fermentations in both IBS and healthy donors. Fermentation of sourdough (type C) bread led to a significant increase in concentrations of butyrate. By focusing on faecal short-chain fatty acids (SCFAs) as the major end product of bacterial metabolism in the human large intestine, researchers have shown that SCFAs were increased in diarrhoea-predominant IBS patients and decreased in constipation-predominant IBS patients. However, another study reported the conflicting finding that SCFAs are decreased in diarrhoea-predominant IBS patients, suggesting that it is necessary to conduct a broader analysis of faecal microbiota, full profiles of organic acids and simultaneous GI symptoms in IBS patients [44].

Nevertheless, the suggested link between the SCFAs profile and GI symptoms can be discussed in the light of the contrasting biological activities of the SCFAs [45]. Acetate is a known chemical irritant, and at high concentrations is used to induce mucosal lesions and abdominal cramps in experimental animals, while butyrate is considered as protective and able to dose-dependently reduce abdominal pain in humans *in vivo* [46]. Tana and co-workers [46] suggest that altered intestinal microbiota contributes to the symptoms of IBS through increased levels of organic acids. Furthermore, IBS patients with high acetic acid or propionic acid levels presented more severe symptoms, impaired quality of life and negative emotions. These results are in accordance with the concept that the gut microbiota influences the sensory, motor and immune system of the gut and interacts with higher brain centers [44–50]. Furthermore, metabolomic analysis of bread fermentation by gut bacteria did not distinguish between the different types of bread, although they all produced larger amounts of SCFAs compared to controls, as expected. Ethanol production was only consistently measured in control patients while only one IBS volunteer out of three was able to produce ethanol from bread fermentation. Ethanol is usually further metabolised to acetate but we could not detect any significant difference in acetate production between IBS and control patients and therefore conclude that the absence of ethanol in IBS patients could not be explained by an increased metabolism to acetate. However, sulphate-reducing bacteria can convert ethanol to acetate, and this could therefore have occurred concomitant with the production of higher organic acids.

*In vitro* gas production was determined in non pH- controlled, 36 h faecal static batch culture tubes. In general, the IBS donors showed higher rates of gas production and total gas compared to healthy donors. Similarly, the rates of gas production for type A and B breads were almost identical in IBS and healthy donors. Sourdough (type C) bread produced significantly lower cumulative gas after 15 h fermentation compared to the other types. This was also observed for the rate of gas production.

To conclude, significant changes were observed in the bacterial populations with sourdough (type C) bread, including lower numbers of sulphate-reducing bacteria, i.e., *Desulfovibrionales*, compared to types A and B.

All types of bread generated some gas after 9 h of fermentation and the patterns of gas production were similar for type A and B breads but were both significantly higher than for type C in IBS donors. In addition, an increase in the concentration of butyrate was the main impact of all breads on the overall SCFA production.

These findings suggest that sourdough products may be advantageous for patients suffering from IBS. This study provides findings supporting the utilization of breads fermented by the traditional long fermentation and sourdough with a positive effect on the composition and metabolic profile of the human intestinal microbiota.
